# Testing the validity of national drug surveys: comparison between a general population cohort and household surveys

**DOI:** 10.1111/add.15371

**Published:** 2021-01-17

**Authors:** Hannah Charles, Jon Heron, Matthew Hickman, Jamie Brown, Lindsey Hines

**Affiliations:** ^1^ National Infection Service Public Health England UK; ^2^ Population Health Sciences, Bristol Medical School University of Bristol Bristol UK; ^3^ Department of Behavioural Science and Health University College London London UK

**Keywords:** ALSPAC, ATS, crime survey for England and wales, illicit drug use, population‐based household surveys, STS, young adults

## Abstract

**Background and Aims:**

There are concerns that national population‐based estimates of illicit drug use are underestimated. We investigated this by comparing estimates of illicit substance use at age 24 from the Crime Survey for England and Wales (CSEW) with a birth cohort (Avon Longitudinal Study of Parents and Children, ALSPAC) and by comparing the Smoking and Alcohol Toolkit Studies (STS/ATS) to ALSPAC.

**Design:**

Cross‐sectional household survey and cross‐sectional data from one wave of a longitudinal birth cohort.

**Setting:**

England and Wales.

**Participants:**

Young adults aged 23–25 reporting on substance use in 2017 to CSEW (*n* = 1165), ALSPAC (*n* = 3389) and STS/ATS (*n* = 950).

**Measurements:**

Lifetime and past‐year illicit drug use, smoking status and hazardous drinking at age 24.

**Findings:**

The 2017 CSEW estimate of lifetime illicit drug use was 40.6%, compared with 62.8% in ALSPAC (risk difference % [RD%] = 22.2%; 95% CI = 18.9–25.5%; *P* ≤ 0.001). The RD in lifetime use between ALSPAC and the CSEW was 23.2% (95% CI = 20.0–26.4%) for cannabis, 16.9% (95% CI = 14.4–19.4%) for powder cocaine and 24.8% (95% CI = 22.6–27.0%) for amphetamine. Past‐year drug use was 16.4% in CSEW, compared with 36.7% in ALSPAC (RD% = 20.3%; 95% CI = 17.6–23.0%; *P* ≤ 0.001). For past‐year substance use, the RD between ALSPAC and the CSEW was 15.4% (95% CI = 12.9–17.9%) for cannabis, 14.8% (95% CI = 13.0%–16.6%) for powder cocaine and 15.9% (95% CI = 14.5–17.4%) for amphetamine. Levels of current smoking were similar between STS (27.4%) and ALSPAC (29.4%). Hazardous drinking was substantially higher in ALSPAC (60.3%) than the ATS (32.1%; RD% = 28.2%; 95% CI = 24.8–31.6%; *P* ≤ 0.001).

**Conclusions:**

The Avon Longitudinal Study of Parents and Children provides one source of validation for measurements of drug use in government household surveys and indicates that illicit drug use may be underestimated in the Crime Survey for England and Wales.

## INTRODUCTION

Globally, substance misuse contributes to 27.8 million disability adjusted life years (DALYs) [1]. Exposure to illicit drugs during adolescence is associated with increased risk of lifetime drug dependence, mental health problems, injury, poorer educational performance [2, 3] and negative socioeconomic outcomes [3, 4, 5].

Reliable measures of illicit drug use are vital for developing effective policy and treatment programmes [6]. It can be practically and methodologically challenging to accurately determine the patterns and trends of illicit drug use through population‐based household surveys [7], and illegality may discourage accurate reporting [8, 9]. Different methodologies may provide insight into the accuracy of population‐based survey estimates of drug use. The Avon Longitudinal Study of Parents and Children (ALSPAC), a large United Kingdom (UK) birth cohort, has a long‐standing relationship between participants and researchers. Participants report a high level of trust in the study [10], which may facilitate accurate reporting of illicit drug use. We compare illicit drug use data from the ALSPAC birth cohort with the population‐based household Crime Survey for England and Wales (CSEW). We compared tobacco and alcohol use in ALSPAC with national Smoking and Alcohol Toolkit Studies (STS/ATS) as a negative control [11] to distinguish whether any differences observed between CSEW and ALSPAC were because of differences in the confounding structure of these populations that generally affected the level of reported use of both legal and illicit substances, or whether differences may be attributable to participant report bias resulting from the illicit nature of the drug use [12]. We expect the prevalence of smoking and alcohol use in the STS/ATS to be similar in ALSPAC.

## METHODS

### Study design

The ALSPAC is a UK population‐based birth cohort. Pregnant women residing in the former Avon Health Authority in South‐West England, who had an expected delivery date between 1 April 1991 and 31 December 1992, were invited to participate. Of their offspring, 13 988 children were alive at age 1. Detailed methods are reported elsewhere [13, 14], with a fully searchable data dictionary available on the ALSPAC study website. Ethical approval for the study was obtained from the ALSPAC Ethics and Law Committee and the local research ethics committees. Study data were collected and managed using REDCap electronic data capture tools hosted at the University of Bristol [15, 16] following participants privately completing electronic computer‐assisted questionnaires.

The CSEW is a household, population‐based survey [17] conducted face‐to‐face in a representative sample of ~35 000 households in England and Wales [18]. Electronic computer‐assisted questionnaires are used for questions concerning illicit drug use [18]. The 70% response rate for the CSEW is relatively high compared to other household surveys [19].

The STS and the ATS are national household surveys conducted by Ipsos MORI to monitor tobacco and alcohol use behaviours of adults in England. These involve monthly face‐to‐face surveys [20]. Every month, ~1700 adults over the age of 16 complete a computer‐assisted survey [21]. The sampling is a hybrid between random probability and simple quota and appears to result in a sample representative of the population on smoking prevalence and consumption and sociodemographic characteristics [22, 23]. However, because of this response, rate cannot be calculated due to lack of a definitive gross sample [24].

### Study population

**Figure 1:**
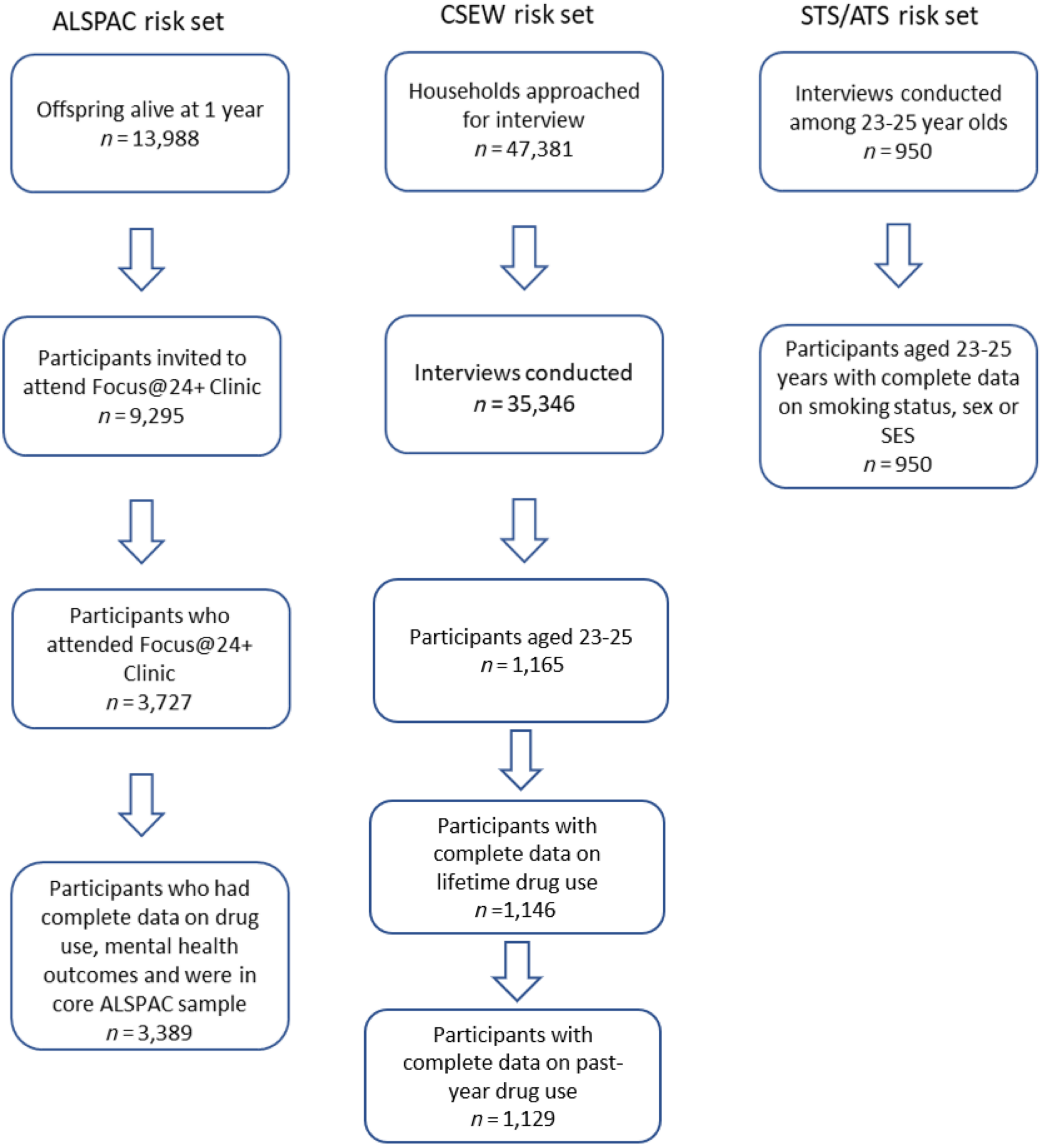
Risk set flow diagrams for each data set. ALSPAC = Avon Longitudinal Study of Parents and Children; CSEW = Crime Survey for England and Wales; STS/ATS = Smoking and Alcohol Toolkit Studies

Figure 1 shows the flow of ALSPAC participants from potential participants to the risk set for this analysis. Participants were those reporting their lifetime and past‐year illicit drug use at a mean age of 24.0 years (SD = 0.8; interquartile range = 23–25 years).

To accurately compare populations, a restricted sample of participants, aged 23–25 years, were derived for the CSEW and STS/ATS. From a sample of 1165, CSEW 2017 participants (mean age = 24, SD = 0.8) with complete data for lifetime and past‐year drug use was available for 1146 and 1129 participants, respectively. STS/ATS sample was 950 (mean age = 24; SD = 0.8).

### Measures

#### Illicit drug use

ALSPAC participants reported their lifetime and past‐year use of cannabis and other illicit drugs (supporting information Data S1 Appendices 1 and 2). To compare to the CSEW data, we only classified ALSPAC participants as reporting lifetime/past‐year illicit drug use if they had reported any use of cannabis, cocaine, crack, amphetamine, hallucinogens and opioids. Any participants who responded ‘no use’ to all of these drugs, or who had reported no use for 9 of 10 of the full complement of ALSPAC drug items (supporting information Data S1 Appendix 1) but had missing data for one remaining item, were coded ‘no use’.

In the CSEW, participants reported on lifetime and past‐year use of cannabis and 11 other drugs (supporting information Data S1 Appendices 1 and 2). Participants were coded as reporting lifetime/past‐year illicit drug use if they had reported any of these drugs within the respective time period.

#### Alcohol consumption

In both ALSPAC and the ATS, participants reported on alcohol use by completing the Alcohol Use Disorders Identification Test for Consumption (AUDIT‐C). AUDIT‐C score >4 was the cut off for hazardous alcohol consumption [25].

### Smoking status

In both ALSPAC and the STS, participants reported current tobacco use. ALSPAC participants reporting smoking in the past 30 days were classified as current smokers. In the STS, participants were defined as current smokers if they reported any cigarette smoking at the time of questioning.

### Sociodemographic variables

Not in education, employment or training (NEET) was reported in ALSPAC and CSEW. Socioeconomic status (SES) was assessed using parent occupation in ALSPAC, coded to a binary variable of high/medium (professional, managerial or skilled occupations) or low (semi‐skilled or manual occupations). In STS/ATS, low SES was defined as chief income earner on state pensions/casual or lowest grade workers/unemployed/claiming state benefits.

### Statistical analysis

All analyses were conducted using STATA version 15.1. Differences between ALSPAC and the household surveys (CSEW, STS and ATS) were tested using two‐sample test of proportions.

### Sample Weighting

Inverse probability weighting (IPW) was applied to the ALSPAC sample. Details are provided in supporting information Data S1 Appendix 3. The inverse of the predicted probabilities were subsequently used as regression weights to adjust the analysis sample to be representative of the 9295 participants invited to the data collection session.

The CSEW data had household weights and individual weights applied to ensure representativeness to the 2011 census population and to account for missing data by adjusting for differential non‐response [18, 26]. In this restricted sample (aged 23–25), 2–3% of participants were missing data on drug use.

STS/ATS data were weighted to match the English population on age, social grade, region, tenure, ethnicity and working status within sex. Data on smoking were complete, and three participants were missing for alcohol use.

These analyses were not pre‐registered, therefore the results should be considered exploratory.

## RESULTS

Table 1 shows there were slight differences in gender distribution and social position. There were comparable numbers of women in the ALSPAC sample compared to the CSEW, yet more than the STS/ATS; and lower proportion of ALSPAC participants in lower social class or NEET than the STS/ATS or the CSEW, respectively.

**Table 1:** Sociodemographic characteristics for ALSPAC, CSEW and STS/ATS samples

*Sociodemographic*	*ALSPAC n = 3389 % (95% CI)*	*CSEW n = 1165 %*	*RD% (95% CI) P value*	*Smoking Toolkit Study and Alcohol Toolkit Study n = 950 %*	*RD% (95% CI) P value*
Female	51.8 (49.9–53.7)	53 (50.0–55.9)	−1.2 (−4.5–2.2) *P* = 0.556	47.4 (44.1–50.6)	4.4 (0.8–8.0) *P* = 0.008
Not in education, employment or training (NEET)	8.4 (7.4–9.6)	4 (3.0–5.3)	4.4 (2.9–5.9) *P* ≤ 0.001	–	–
Parents' lower socioeconomic status	4.2 (3.4–5.3)	–	–	8.7 (7.0–10.7)	−4.5 (−6.4–2.6) P ≤ 0.001

ALSPAC = Avon Longitudinal Study of Parents and Children; CSEW = Crime Survey for England and Wales; RD% = risk difference % (ALSPAC as comparator); STS/ATS = Smoking and Alcohol Toolkit Studies.

Table 2 shows the prevalence comparison of past‐year and lifetime use of different illicit drugs. Past‐year illicit drug use was reported by 36.7% of the ALSPAC sample at age 24, compared to 16.4% in the CSEW, a difference of 20.3% (95% CI = 17.6–23.0%, *P* ≤ 0.001); and in the ALSPAC sample, 62.8% of the ALSPAC sample reported any lifetime illicit drug use at age 24, compared to 40.6% in the CSEW, a difference of 22.2% (95% CI = 19.0–25.5%, *P* ≤ 0.001). The difference in lifetime illicit drug use between ALSPAC and the CSEW was greatest for amphetamine, cannabis and powder cocaine, where the difference in prevalence was 24.8% (95% CI = 22.6–27.0%, *P* ≤ 0.001), 23.2% (95% CI = 20.0–26.4%, *P* ≤ 0.001) and 16.9% (95% CI = 14.4–19.4%, *P* ≤ 0.001) respectively. The difference in past‐year use between ALSPAC and the CSEW was greatest for cannabis, powder cocaine and amphetamine, where the difference in prevalence was 15.4% (95% CI = 12.9–17.9%, *P* ≤ 0.001), 14.8% (95% CI = 13.0–16.6%, P ≤ 0.001) and 15.9% (95% CI = 14.5–17.3%, *P* ≤ 0.001), respectively.

**Table 2:** Prevalence comparison of drug use between ALSPAC and the CSEW

	*Past‐year drug use*			*Lifetime drug use*		
	*ALSPAC Weighted 3389 % (95% CI)*	*CSEW Weighted 1129 %*	*RD% (95% CI) P value*	*ALSPAC Weighted 3389 % (95% CI)*	*CSEW Weighted 1146 %*	*RD% (95% CI) P value*
Any illicit drug use	36.7 (35.0–38.6)	16.4 (14.3–18.7)	20.3 (17.6–23.0) *P* ≤ 0.001	62.8 (61.1–64.6)	40.6 (37.8–43.5)	22.2 (19.0–25.5) *P* ≤ 0.001
Amphetamine	17.0 (15.7–18.4)	1.1 (0.6–1.9)	15.9 (14.5–17.3) *P* ≤ 0.001	32.9 (31.2–34.7)	8.1 (6.6–9.8)	24.8 (22.6–27.0) *P* ≤ 0.001
Anabolic steroids	^a^	0.4 (0.01–1.0)	–	^a^	1.3 (0.7–2.1)	–
Cannabis	29.2 (27.5–31.0)	13.8 (11.8–15.9)	15.4 (12.9–17.9) *P* ≤ 0.001	60.5 (58.7–62.3)	37.3 (34.5–40.2)	23.2 (20.0–26.4) *P* ≤ 0.001
Powder cocaine	19.6 (18.2–21.2)	4.8 (3.6–6.2)	14.8 (13.0–16.6) *P* ≤ 0.001	30.8 (29.1–32.6)	13.9 (11.9–16.0)	16.9 (14.4–19.4) *P* ≤ 0.001
Crack cocaine	0.2 (0.0–0.5)	0.0 (0.0–0.3)	0.2 (0.0–0.4) *P* = 0.127	1.0 (0.6–1.7)	0.9 (0.4–1.6)	0.1 (−0.5–0.7) *P* = 0.764
Ecstasy	^b^	3.6 (2.6–4.8)	–	^b^	11.1 (9.3–13.0)	–
Hallucinogens	7.3 (6.4–8.3)	1.5 (0.9–2.4)	5.8 (4.7–6.9) *P* ≤ 0.001	18.1 (16.7–19.7)	6.8 (5.4–8.4)	11.3 (9.3–13.2) *P* ≤ 0.001
Inhalants	0.7 (0.5–1.2)	^a^	–	2.6 (2.0–3.3)	^a^	–
Injecting drug use	0.2 (0.0–0.4)	^a^	–	0.3 (0.2–0.6)	^a^	–
Ketamine	^b^	1.1 (0.6–1.9)	–	^b^	5.1 (3.9–6.5)	–
Mephedrone	^b^	0.4 (0.1–1.0)	–	^b^	5.4 (4.2–6.9)	–
Nitrous oxide	20.6 (19.2–22.1)	^a^	–	45.6 (43.8–47.5)	^a^	–
Opioids	2.7 (2.2–3.3)	0.0 (0.0–0.3)	2.7 (2.2–3.2) *P* ≤ 0.001	5.0 (4.2–5.9)	0.9 (0.4–1.6)	4.1 (3.2–5.0) *P* ≤ 0.001
Sedatives/tranquilisers	5.8 (5.0–6.7)	1.1 (0.6–1.9)	4.7 (3.7–5.7) *P* ≤ 0.001	11.6 (10.4–12.9)	3.5 (2.5–4.7)	8.1 (6.6–9.6) *P* ≤ 0.001

ALSPAC = Avon Longitudinal Study of Parents and Children; CSEW = Crime Survey for England and Wales; RD% = Risk difference % (ALSPAC as comparator).

^a^ No equivalent in this sample.

^b^ Included in another category in this sample (see supporting information Data S1 Appendix 1).

It must be noted that the amphetamine category in ALSPAC included 3,4‐methylenedioxymethamphetamine (MDMA), whereas CSEW listed ecstasy as a separate item. This may contribute to some of the difference in the results comparing amphetamine use. However, the proportion of participants in CSEW endorsing ecstasy use (11.1% and 3.6% for lifetime and past‐year use, respectively) indicates this discrepancy in categorisation would not completely account for the differences observed.

In ALSPAC, 60.3% of participants reported hazardous drinking at age 24, compared to 32.1% in the ATS, a difference of 28.2% (95% CI = 24.8–31.6%, *P* ≤ 0.001). There was no difference in tobacco use between ALSPAC and STS (29.4% and 27.4%, respectively) (Table 3).

**Table 3:** Substance use comparison between ALSPAC and STS/ATS

	*ALSPAC n = 3389 % (95% CI)*	*Smoking Toolkit Study and Alcohol Toolkit Study n = 950 % (95% CI)*	*RD% (95% CI) P value RD% (95% CI)*
Hazardous alcohol use	60.3 (58.6–62.2)	32.1 (29–35)	28.2 (24.8–31.6) *P* ≤ 0.001
Current tobacco use	29.4 (27.7–31.2)	27.4 (25–30)	2.0 (−1.2–5.2) *P* = 0.23

ALSPAC = Avon Longitudinal Study of Parents and Children; RD% = Risk difference % (ALSPAC as comparator); STS/ATS = Smoking and Alcohol Toolkit Studies.

## DISCUSSION

By comparing the CSEW household survey to data from a birth cohort, we have found the CSEW may significantly underestimate drug use in the general population. The absolute difference between past‐year and lifetime illicit drug use in these two different samples were >20%, corresponding to a greater than twofold difference. In our negative control, there was no difference between ALSPAC and the STS/ATS in tobacco use, but differences were observed in hazardous drinking.

Different research methodologies can affect estimates of illicit drug use [27]. It has been previously reported the CSEW underestimates the prevalence of drug use in England and Wales [28]. Weisner and colleagues reported a disparity of 31% between the recent drug use reported in a household sample compared to other survey methods [29]. Although the use of computerised surveys in the CSEW provides increased anonymity, the inclusion of these questions in the context of experience of crime may reduce the likelihood of accurate illicit substance use disclosure [30]. ALSPAC is a longitudinal study where participant rapport and trust have been built up over time. ALSPAC's strict procedures of anonymity and confidentiality are made explicitly clear to participants [31]. Qualitative research reported participants were trusting and had faith in ALSPAC and ‘considered themselves part of the ALSPAC team’ [10]. This may help to promote more accurate and honest drug use self‐reporting.

The differences in hazardous alcohol use between ALSPAC and the ATS (included as a negative control) were large, but it has been suggested ATS may underestimate hazardous alcohol consumption [32, 33]. Although Local Alcohol Profiles [34] suggest Bristol is slightly higher than the England average for alcohol related harm, there is not an exceptional regional difference. The 2014 Adult Psychiatric Morbidity Survey (APMS) reported 19.7% of participants had an AUDIT score >7, compared to 13% reported using the ATS—a risk difference of 7%, or 50%, as opposed to the 28% or >80%, we find in hazardous use between the ATS and ALSPAC [32, 33]. The ATS may underestimate alcohol consumption compared with ALSPAC and APMS if respondents feel less comfortable disclosing their alcohol use with the market research company, Ipsos MORI.

### Strengths and limitations

All of the data collection methodologies have incomplete response rates and rely on self‐report. There is no evidence drug and alcohol use is markedly different in Bristol and Avon than elsewhere in England [35, 36], but common mental health disorders may be more prevalent in the South West of England [36]. The differences in reported use are greater than random error. ALSPAC response rates for young adults are lower than earlier data collection points [14] and therefore there is substantial missing data from the Focus@24 + clinic (see Fig. **1**). The original ALSPAC sample was representative of Avon but not the United Kingdom, and sociodemographic factors such as affluence, sex and ethnicity impact on response rates and therefore Caucasian people and those from more affluent backgrounds are over‐represented compared to the general population [14]. However, IPW was used to make the ALSPAC sample (*n* = 3389) representative of the 9295 people invited to the clinic, indicating selection bias is unlikely to underlie present results.

### Implications

It is important to ensure the CSEW and other government household surveys accurately measure drug use. The level of accuracy has implications for the ability of drug policies, prevention and treatment interventions to adequately meet the need of the population and accurate estimates are required to improve the evidence‐base in the field. ALSPAC provides one source of validation and indicates illicit drug use may be underestimated in the CSEW. However, as ALSPAC participants are drawn from one region of the United Kingdom, we urgently need to expand this work to other birth cohort studies to further validate the results.

## Declaration of interests

There are no declarations of competing interests.

## Funding

The UK Medical Research Council and Wellcome (102215/2/13/2) and the University of Bristol provide core support for ALSPAC. This publication is the work of the authors and H.C., J.H., M.H., J.B. and L.H. will serve as guarantors for the contents of this paper. CRUK‐funded (C1417/A22962) Smoking and Alcohol Toolkit Study for sharing data on prevalence of smoking and hazardous drinking in England. The work was undertaken with the support of The Centre for the Development and Evaluation of Complex Interventions for Public Health Improvement (DECIPHer), a UKCRC Public Health Research Centre of Excellence. Joint funding (MR/KO232331/1) from the British Heart Foundation, Cancer Research UK, Economic and Social Research Council, Medical Research Council, the Welsh Government and the Wellcome Trust, under the auspices of the UK Clinical Research Collaboration, is gratefully acknowledged. We also acknowledge funding from the NIHR School of Public Health Research, NIHR Health Protection Research Unit in Behavioural Science and Evaluation and NIHR BRC at Bristol. The MRC and Alcohol Research UK (MR/L022206/1) funded data collection and also supports J.H. L.H. is funded by The Wellcome Trust.

## Author contributions

**Hannah Charles:** Conceptualization; data curation; formal analysis; investigation; methodology. **Jon Heron:** Data curation; formal analysis; methodology. **Matthew Hickman:** Conceptualization; methodology; supervision. **Jamie Brown:** Investigation; methodology. **Lindsey Hines:** Conceptualization; data curation; formal analysis; investigation; methodology; project administration; supervision.

## Supporting information

**Appendix 1.** Comparison of illicit drugs measured in ALSPAC and CSEW
**Appendix 2.** Drug use questions in ALSPAC and the CSEW
**Appendix 3.** Details of sample weights applied to ALSPAC data
