# Supplementary material for: Testing the validity of national drug surveys: comparison between a general population cohort and household surveys
Source: Addiction. 2021 Jan 17;116(8):2076–83. doi: 10.1111/add.15371 (PMC8359398; doi:10.1111/add.15371)
Supplement: Supplementary file 1 — Appendix 1. Comparison of illicit drugs measured in ALSPAC and CSEW Appendix 2. Drug use questions in ALSPAC and the CSEW Appendix 3. Details of sample weights applied to ALSPAC data [file ADD-116-2076-s001.docx]

**Appendices**

Page 1 Appendix 1: Comparison of illicit drugs measured in ALSPAC and CSEW

Page 2 Appendix 2: Drug use questions in ALSPAC and the CSEW

Page 7 Appendix 3: Details of sample weights applied to ALSPAC data

**Appendix 1: Comparison of illicit drugs measured in ALSPAC and CSEW**

| **ALSPAC** | **CSEW** |
| --- | --- |
| Amphetamine-type stimulants (speed, base, diet pills, ecstasy, MDMA, GHB, 2CB, 2CI, Mcat, Mephedrone) | Amphetamine (amphetamines, methamphetamine) |
| † | Anabolic steroids |
| Cannabis | Cannabis |
| (Powder) Cocaine (Charlie, ‘c’, coke) | (Powder) Cocaine |
| Crack cocaine (rock, stone) | Crack cocaine |
| * | Ecstasy |
| Hallucinogens (LSD, acid, mushrooms, PCP, ketamine, Special K, N-Bomb) | Hallucinogens (LSD, magic mushrooms) |
| Inhalants (glue, petrol, paint thinner) | † |
| Injected illicit drugs | † |
| * | Ketamine |
| * | Mephedrone |
| Nitrous oxide (laughing gas) | † |
| Opiates (heroin, morphine, methadone, codeine) | Opiates (heroin, methadone) |
| Sedatives or sleeping pills (Valium, rohypnol) | Tranquilisers |

†No equivalent in this sample

*Included in another category in this sample

**Appendix 2: Drug use questions in ALSPAC and the CSEW**

**ALSPAC questions on drug use at age 24**

*The next questions are about other drugs that people sometimes take. Your answers will be kept confidentially, like any medical notes. The information you give will be treated in strict confidence and will only be used for research purposes.*

In your life, which of the following substances have you ever used? (Non-medical use only.)

Cocaine (Charlie, 'c', coke, etc.)

Have you tried it in the last 12 months?

Crack (rock, stone, etc.)

Have you tried it in the last 12 months?

Amphetamine-type stimulants (speed, base, diet pills, ecstacy, MDMA, GHB, 2CB, 2CI, Mcat, Mephedrone etc.)

Have you tried it in the last 12 months?

Nitrous oxide (laughing gas)

Have you tried it in the last 12 months?

Other inhalants (glue, petrol, paint thinner etc.)

Have you tried it in the last 12 months?

Sedatives or sleeping pills (Valium, Rohypnol, etc.)

Have you tried it in the last 12 months?

Hallucinogens (LSD, acid, mushrooms, PCP, ketamine, Special K, N-Bomb etc.)

Have you tried it in the last 12 months?

Opioids (heroin, morphine, methadone, codeine etc.)

Have you tried it in the last 12 months?

Injected illicit drugs

Have you tried it in the last 12 months?

**CSEW (2016/2017) questions on illicit drug use**

1. Have you EVER taken AMPHETAMINES (SPEED, WHIZZ, UPPERS, BILLY, SULPHATE,

CRANK, PASTE), even if it was a long time ago?

- Yes
- No
- Never heard of it
- Don't want to answer

1. Have you EVER taken METHAMPHETAMINE (CRYSTAL METH, ICE, GLASS, TINA, YABA)

even if it was a long time ago?

- Yes
- No
- Never heard of it
- Don't want to answer

1. Have you EVER taken CANNABIS (MARIJUANA, GRASS, HASH, GANJA, BLOW, DRAW,

SKUNK, WEED, SPLIFF, DOPE), even if it was a long time ago?

- Yes
- No
- Never heard of it
- Don't want to answer

1. Have you EVER taken COCAINE POWDER (COKE, CHARLIE) even if it was a long time ago?

- Yes
- No
- Never heard of it
- Don't want to answer

1. Have you EVER taken CRACK COCAINE (BASE, ROCK, STONES) even if it was a long time

ago?

- Yes
- No
- Never heard of it
- Don't want to answer

1. Have you EVER taken ECSTASY ('E', MDMA), even if it was a long time ago?

- Yes
- No
- Never heard of it
- Don't want to answer

1. Have you EVER taken HEROIN (SMACK, 'H', BROWN), even if it was a long time ago?

- Yes
- No
- Never heard of it
- Don't want to answer

1. Have you EVER taken LSD/ACID (TRIPS), even if it was a long time ago?

- Yes
- No
- Never heard of it
- Don't want to answer

1. Have you EVER taken MAGIC MUSHROOMS, even if it was a long time ago?

- Yes
- No
- Never heard of it
- Don't want to answer

1. Have you EVER taken METHADONE or PHYSEPTONE (not prescribed by a doctor or other

healthcare professional), even if it was a long time ago?

- Yes
- No
- Never heard of it
- Don't want to answer

1. Have you EVER taken TRANQUILLISERS (TEMAZEPAM, VALIUM, ROOFIES, JELLIES) (not

prescribed by a doctor or other healthcare professional), even if it was a long time ago?

- Yes
- No
- Never heard of it
- Don't want to answer

1. Have you EVER taken ANABOLIC STEROIDS (STEROIDS) (not prescribed by a doctor or other

healthcare professional), even if it was a long time ago?

- Yes
- No
- Never heard of it
- Don't want to answer

1. Have you EVER taken KETAMINE (K, SPECIAL K, VITAMIN K) (not prescribed by a doctor or

other healthcare professional), even if it was a long time ago?

- Yes
- No
- Never heard of it
- Don't want to answer

1. Have you EVER taken MEPHEDRONE (Meow Meow, MCAT, Bubble, Drone, Meph, 4MMC)

even if it was a long time ago?

- Yes
- No
- Never heard of it
- Don’t want to answer

**18.3 WHETHER RESPONDENT HAS TAKEN DRUGS IN LAST 12 MONTHS**

1. In the last 12 MONTHS have you taken AMPHETAMINES (SPEED, WHIZZ, UPPERS, BILLY,

SULPHATE, CRANK, PASTE)?

- Yes
- No
- Don't want to answer

1. In the last 12 MONTHS have you taken METHAMPHETAMINE (CRYSTAL METH, ICE, GLASS,

TINA, YABA)?

- Yes
- No
- Don't want to answer

1. In the last 12 MONTHS have you taken CANNABIS (MARIJUANA, GRASS, HASH, GANJA,

BLOW, DRAW, SKUNK, WEED, SPLIFF, DOPE)?

- Yes
- No
- Don't want to answer

1. In the last 12 MONTHS have you taken COCAINE POWDER (COKE, CHARLIE)?

- Yes
- No
- Don't want to answer

1. In the last 12 MONTHS have you taken CRACK COCAINE (BASE, ROCK, STONES)?

- Yes
- No
- Don't want to answer

1. In the last 12 MONTHS have you taken ECSTASY ('E', MDMA)?

- Yes
- No
- Don't want to answer

1. In the last 12 MONTHS have you taken HEROIN (SMACK,'H', BROWN)?

- Yes
- No
- Don't want to answer

1. In the last 12 MONTHS have you taken LSD/ACID (TRIPS)?

- Yes
- No
- Don't want to answer

1. In the last 12 MONTHS have you taken MAGIC MUSHROOMS?

- Yes
- No
- Don't want to answer

1. In the last 12 MONTHS have you taken METHADONE or PHYSEPTONE (not prescribed by a

doctor or other healthcare professional)?

- Yes
- No
- Don't want to answer

1. In the last 12 MONTHS have you taken TRANQUILLISERS (TEMAZEPAM, VALIUM, ROOFIES,

JELLIES) (not prescribed by a doctor or other healthcare professional)?

- Yes
- No
- Don't want to answer

1. In the last 12 MONTHS have you taken ANABOLIC STEROIDS (STEROIDS) (not prescribed by

a doctor or other healthcare professional)?

- Yes
- No
- Don't want to answer

1. In the last 12 months have you taken KETAMINE (K, SPECIAL K, VITAMIN K) (not prescribed by

a doctor or other healthcare professional)?

- Yes
- No
- Don't want to answer

1. In the last 12 MONTHS have you taken MEPHEDRONE (Meow Meow, MCAT, Bubble, Drone,

Meph, 4MMC)?

- Yes
- No

Don’t want to answer

**Appendix 3: Details of sample weights applied to ALSPAC data**

Measures included in weights

The following variables collected via postal questionnaires filled in by the mothers during pregnancy were included in the sample weights: mother’s educational level (university degree, further education, compulsory school qualifications, or no qualifications); housing tenure (rented, owner occupied); housing crowding; housing defects during pregnancy; financial difficulties during pregnancy; partner affection during pregnancy; partner cruelty during pregnancy; partner support during pregnancy; emotional and practical support from social network during pregnancy.

Participant gender was included.

Weight model fit

As IPW is sensitive to very large numbers, the IPWs were truncated to the 95% percentile (4.80). Hosmer-Lemeshow chi2 was 5.25.
